# Hedgehog signaling controls segmentation dynamics and diversity via *msx1* in a spider embryo

**DOI:** 10.1126/sciadv.aba7261

**Published:** 2020-09-09

**Authors:** Yasuko Akiyama-Oda, Hiroki Oda

**Affiliations:** 1 Laboratory of Evolutionary Cell and Developmental Biology, JT Biohistory Research Hall, Takatsuki, Osaka, Japan.; 2 Microbiology and Infection Control, Osaka Medical College, Takatsuki, Osaka, Japan.; 3 Department of Biological Sciences, Graduate School of Science, Osaka University, Toyonaka, Osaka, Japan.

## Abstract

Hedgehog (Hh) signaling plays fundamental roles in animal body patterning. Understanding its mechanistic complexity requires simple tractable systems that can be used for these studies. In the early spider embryo, Hh signaling mediates the formation of overall anterior-posterior polarity, yet it remains unclear what mechanisms link the initial Hh signaling activity with body axis segmentation, in which distinct periodic stripe-forming dynamics occur depending on the body region. We performed genome-wide searches for genes that transcriptionally respond to altered states of Hh signaling. Characterization of genes negatively regulated by Hh signaling suggested that *msx1*, encoding a conserved transcription factor, functions as a key segmentation gene. Knockdown of *msx1* prevented all dynamic processes causing spatial repetition of stripes, including temporally repetitive oscillations and bi-splitting, and temporally nonrepetitive tri-splitting. Thus, Hh signaling controls segmentation dynamics and diversity via *msx1*. These genome-wide data from an invertebrate illuminate novel mechanistic features of Hh-based patterning.

## Teaser

A genome-wide study of Hedgehog signaling reveals a previously unknown mechanism of segmentation in a chelicerate arthropod.

## INTRODUCTION

Hedgehog (Hh) signaling is one of the most important signaling pathways in animal development. It mediates the formation of polarity and patterning in cellular fields of various animal tissues, such as *Drosophila* segments and imaginal discs (*1*–*4*) and the vertebrate neural tube and limb buds (*5*, *6*). The Hh signaling pathway is composed of tens of components that function as complex molecular networks with positive and negative feedbacks. The main components of this pathway include the secreted signaling protein Hh, its receptor Patched (Ptc), which also acts as a negative regulator, the membrane signal transducer Smoothened (Smo), and the signaling effector transcription factor Cubitus interruptus (Ci, Gli in vertebrates) (*7*). Gradients of concentration- and time-dependent Hh signaling activities are thought to provide positional values to cells in the entire tissue field, leading to the establishment of discrete domains and repetitive stripes (*8*–*11*). In this process, transcriptions of genes respond to different thresholds of the graded signaling activities, which specifies various spatial regions in the tissue field (*10*, *11*). The concentration-dependent effects of diffusible Hh on cell fate specification support the categorization of this molecule as a morphogen, which is assumed in the French flag model (*12*). However, the time dependency of the signaling effects and the positive and negative feedbacks reflect the mechanistic complication of the system (*10*, *11*). Further complications arise from the dynamics of tissue fields, where Hh signaling functions for patterning. Cell proliferation, cell lineages, cell behaviors, and spatial organization may have limited the understanding of the patterning activities of Hh signaling.

**Fig. 1:**
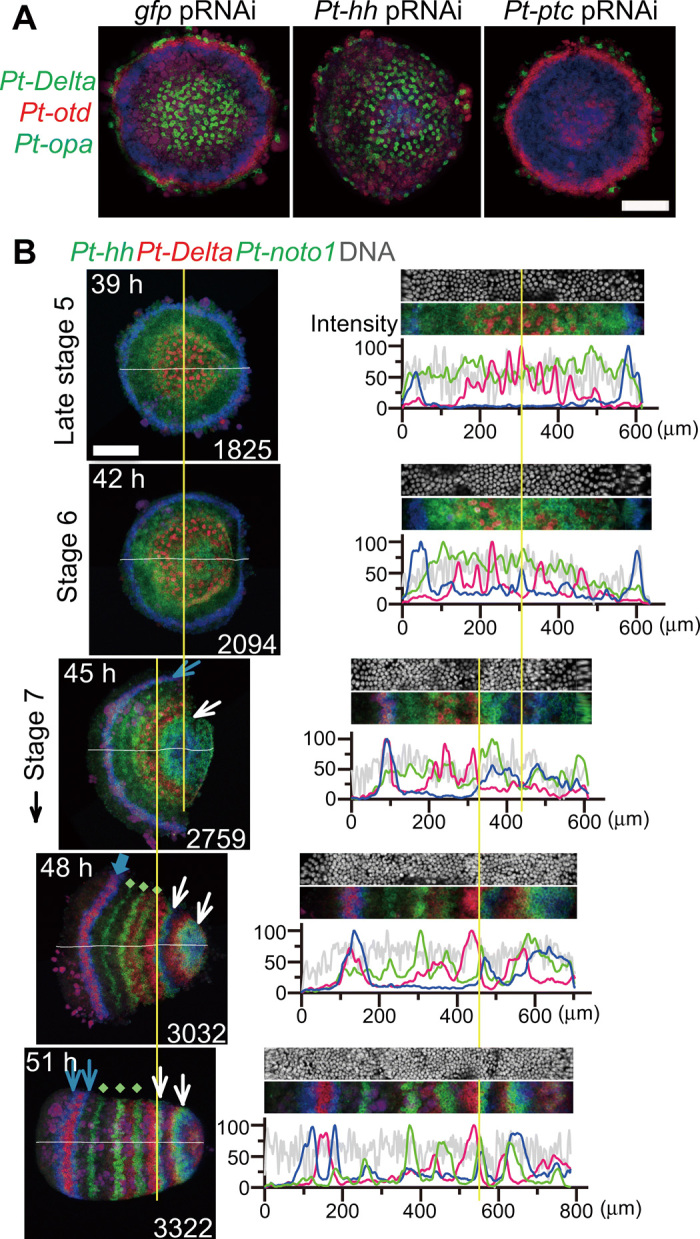
Early patterning in the spider embryo. (**A**) Polarity phenotypes of late stage 5 *gfp*^pRNAi^, *Pt-hh*^pRNAi^, and *Pt-ptc*^pRNAi^ germ discs stained by FISH for *Pt-Delta* (green), *Pt-otd* (red), and *Pt-opa* (blue) transcripts. (**B**) Stripe pattern development during the germ disc to germ band transition. Left: Semi-flat preparations of sibling embryos stained for *Pt-hh* (blue), *Pt-Delta* (red), and *Pt-noto1* (green) transcripts and DNA (shown only in right panels). Approximate times AEL (top left) and surface cell counts (bottom right). Right: Signal intensity profiles in the surface cell layer along the midline (white line, left; averaged across 20-μm width); extracted images (80-μm wide) are shown together. Yellow lines refer to the center of the germ disc and the likely corresponding position (39 to 45 hours) and the posterior limit of the *Pt-Delta* L4 stripe (45 to 51 hours). Blue arrows indicate the anterior *Pt-hh* stripe(s) that undergo traveling and splitting (45 to 48 hours), green dots indicate *Pt-noto1* stripes that appear simultaneously in the thorax (48 and 51 hours), and white arrows mark *Pt-hh* stripes generated through oscillations for L4 and O1 segments (45 to 51 hours). Scale bars, 200 μm.

The early embryo of the spider *Parasteatoda tepidariorum* (synonym, *Achaearanea tepidariorum*) (*13*) forms a radially symmetrical germ disc in a monolayer at the spherical surface, where concentric gene expression patterns occur, reflecting the polarity of the anterior-posterior (AP) axis. These patterns, which are French flag–like, are established by a mechanism mediated by Hh signaling (*14*) (Fig. 1A). The spider *hh* homolog, *Pt-hh* (aug3.g4322, formerly called *At-hh*), is zygotically expressed in cells of the future anterior that is located opposite to the embryonic pole (the center of the forming germ disc) (*14*) and the rim of the formed germ disc (Fig. 1B). Following a symmetry-breaking event (*15*, *16*), the germ disc undergoes a relatively simple planar remodeling and is transformed into an axially growing germ band, in which spatially repetitive stripes of gene expression associated with body axis segmentation occur in three different modes depending on the region of the body (Fig. 1B) (*17*). The repetitive stripes fail to be formed in *Pt-hh* knockdown embryos (*14*). *Pt-hh* is one of the earliest genes that are expressed in repetitive stripes. The anterior *Pt-hh* stripe, which originates from the germ disc rim and travels posteriorly in the nascent germ band, undergoes repeated bi-splitting in the head region (*18*), whereas oscillatory *Pt-hh* expression waves sweep successively in the opisthosoma that is derived from the central region of the germ disc. Striped *Pt-hh* expression in the thoracic region, however, appears substantially later. Instead, *Pt-noto1* (aug3.g15243) stripes simultaneously appear in this region at the earliest timing (*17*). The stripes of *Pt-hh* formed in the germ band (*19*) are presumably associated with the formation of segment polarity. Thus, the early *P. tepidariorum* embryo provides a simple cell-based platform where the patterns reflecting the AP polarity and the repetitive stripes associated with the body segments are formed through Hh signaling–mediated mechanisms. To date, little is known about mechanisms that link the initial Hh signaling activity with the periodic stripe pattern formation.

The segmental stripe pattern along the AP axis is achieved by several distinct modes in arthropods (*20*, *21*); however, the functions of Hh signaling in the earliest step of AP patterning remain unclear in species other than *Parasteatoda*. In the long germ insect *Drosophila*, the hierarchical interactions of maternal, gap, and pair-rule genes, most of which encode transcription factors, simultaneously subdivide the syncytial embryonic field into spatially repetitive units, wherein the polarity is further specified by segment polarity genes including *hh* (*1*–*3*). This segment polarity function seems a conserved feature of arthropods, since the *hh* genes are expressed in the posterior part of each segment in a wide range of arthropods (*22*, *23*). Although the expression of *hh* in the anterior region of the early embryo has been reported in some arthropod species (*1*, *23*), the functional significance of the early *hh* expression remains unclear except that observed in the spider *Parasteatoda*.

Here, we used the spider embryo to investigate spatiotemporal changes in gene expression downstream of Hh signaling. Subsequently, we characterized mechanistic links between formation of the Hh signaling–mediated field polarity and the dynamics and diversity of segmentation-related gene expression.

## RESULTS

### Hh signaling components in the *Parasteatoda* genome

We performed Basic Local Alignment Search Tool (BLAST) searches against the *Parasteatoda* genome and annotated genes for Hh signaling components using the amino acid sequences from *Drosophila*. For those that are not present in the *Drosophila* genome, the sequences of the human were used. The spider genes that had hits with substantial *e*-values were then used reciprocally in BLAST searches against *Drosophila* or human proteins to examine whether the spider genes showed the highest hits to the original proteins. These searches revealed that the *Parasteatoda* genome encodes all core components of the Hh signaling pathways that have been described in *Drosophila* and vertebrates (table S1) (*24*, *25*). Some of these genes, including *hh*, are present in duplicate. Of the two *hh* homologs, only *Pt-hh* (aug3.g4322) is expressed at substantial levels during embryogenesis (*26*). The other relevant genes, such as *patched* (*Pt-ptc*, aug3.g14374; formerly known as *At-ptc*) (*14*), *smoothened* (*Pt-smo*, aug3.g8490; *At-smo*) (*14*), and *cubitus interruptus* (*Pt-ci*, aug3.g13321; *At-ci*) (*18*), are present as single copies.

### Genome-wide searches for genes responding to *Pt-hh* and *Pt-ptc* knockdown

**Fig. 2:**
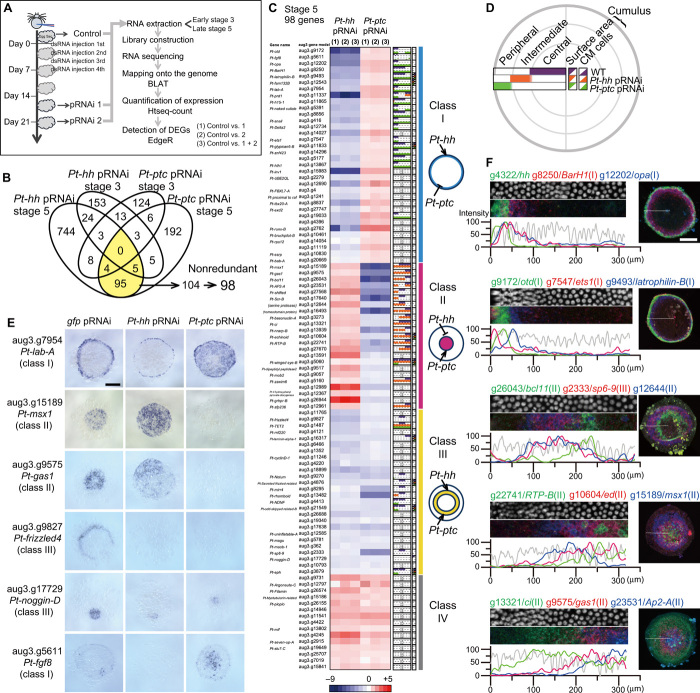
Genome-wide identification and categorization of genes regulated by Hh signaling. (**A**) Strategy for identifying DEGs between untreated and pRNAi-treated embryos. BLAT, Blast-like alignment tool. (**B**) Venn diagram showing the numbers of identified DEGs. DEGs identified in both *Pt-hh* and *Pt-ptc* pRNAi experiments at late stage 5 (yellow) were deduced into 98 nonredundant genes. (**C**) The heatmap showing fold changes (log_2_, the color code at the bottom) of expression levels of DEGs from stage 5 experiments, calculated from three comparisons (1) to (3). The column to the immediate right of the heatmap shows spatial expression ranges along the radius of the late stage 5 germ disc with color-coded horizontal bars and triangles. Gray, unclear staining; n.d., not determined. In the right, four categories of genes, their representative expression pattern in the germ disc, and their regulation by *Pt-hh* and *Pt-ptc* are schematically represented. (**D**) Explanation of the expression color code and expression areas shown in (C). Spatial expression ranges observed in WT, *Pt-ptc*^pRNAi^, and *Pt-hh*^pRNAi^ germ discs are illustrated in purple, orange, and green, respectively. (**E**) Expression of some of the DEGs in late stage 5 pRNAi germ discs. These photos are the same as in data file S5. (**F**) Varied expression profiles of DEGs and *Pt-hh* in late stage 5 WT germ discs. Data and images are shown as in Fig. 1B, except that extracted images are 40-μm wide and along the radius. The numbers in parentheses indicate the classes of the genes. Germ discs are oriented so that the CM cells are to the right. Scale bars, 200 μm (E and F). Photo credit: Yasuko Akiyama-Oda, JT Biohistory Research Hall.

Parental RNA interference (pRNAi)–mediated knockdowns of *Pt-hh* and *Pt-ptc* have been shown to produce contrasting polarity defects with the highest penetrance (Fig. 1A) (*14*), where the jellyfish *green fluorescent protein* (*gfp*) gene served as the control. Combining these knockdowns with RNA sequencing (RNA-seq), we conducted genome-wide searches for genes that transcriptionally responded to altered states of Hh signaling at early stage 3 and late stage 5 to comprehensively understand Hh signaling–mediated patterning in the early spider embryo (Fig. 2A). In early stage 3, the embryo presents morphological asymmetry and expresses *Pt-hh* on the future anterior side, and in late stage 5, the germ disc reveals region-specific gene expressions (*14*). RNA was extracted from eggs laid by the same females before and after double-stranded RNA (dsRNA) injection, and the RNA-seq, genome mapping, and comparison of gene expression levels were performed to identify the differentially expressed genes (DEGs; see Fig. 2A, data files S1 to S4, and Materials and Methods).

To validate the methods, we first looked at mapping data at the *Pt-hh* and *Pt-ptc* loci. At the *Pt-hh* locus, the number of mapped reads were drastically reduced in all *Pt-hh* pRNAi samples, and *Pt-hh* was consistently detected as a DEG at early stage 3 and late stage 5 (data file S3). On the other hand, in *Pt-ptc* pRNAi experiments, *Pt-ptc* itself was detected as a DEG at early stage 3 but not at late stage 5 (data file S4). As *ptc* is a target of Hh signaling and acts as a negative regulator of Hh signaling (*7*), *Pt-ptc* knockdown likely promotes Hh signaling and activates the transcription of *Pt-ptc*. Nonetheless, the previous study showed that *Pt-ptc* transcripts were less detectable in the cytoplasm in *Pt-ptc*^pRNAi^ embryos, indicating that the transcription was active but transcripts were unstable in the cytoplasm (*14*). These data suggested that the state of Hh signaling was expectedly altered by *Pt-hh* and *Pt-ptc* pRNAi.

We also examined mapping data at the loci of known target genes (*14*, *18*). At the loci of *Pt-otd* (aug3.g9172), *Pt-lab-A* (aug3.g7954), and *Pt-opa* (aug3.g12202), numbers of mapped reads were all affected by *Pt-hh* and *Pt-ptc* pRNAi at late stage 5, as expected (fig. S1). These genes were also ranked highly in the list of DEGs (data files S3 and S4), further validating our experiments. In contrast, numbers of reads mapped to the *Pt-Delta* (aug3.g25248) locus were not clearly altered (fig. S1), and this gene was not listed among DEGs in stage 5 experiments (data files S3 and S4), despite our previous observations that expression of *Pt-Delta* in the germ disc is regulated by Hh signaling (Fig. 1A) (*14*). This discrepancy is because additional expression in cells of the yolk side is independent of Hh signaling regulation (*14*, *19*). Together, these results indicated that our list of DEGs included a substantial number of genes that are regulated by Hh signaling.

At a false discovery rate (FDR) threshold of adjusted *P* value < 0.01 in both *Pt-hh* and *Pt-ptc* pRNAi experiments, 104 aug3 gene models were identified as differentially expressed at late stage 5 (Fig. 2B and table S2). However, we found that the 104 gene models included at least six redundancies. Specifically, aug3.g4422 and g22667 sequences were overlapped by more than 750 bp, aug3.g416 and g.417 were derived from the coding region and the 3′ untranslated region of *Pt-snail* (formerly *At.snail*), respectively (*27*). Evidence from reverse transcription polymerase chain reaction (PCR) and rapid amplification of cDNA ends analyses suggested that pairs of gene models aug3.g1485-g1487, aug3.g24611-g27568, aug3.g27747-g27748, and aug3.g407-g22741 were derived from single genes. Thus, the DEG count of 104, for the experiments with late stage 5 embryos, was reduced to 98. On the other hand, only 19 DEGs were identified in early stage 3, and these were not among the 98 late stage 5 DEGs (Fig. 2B). These data suggested that early stage 3 and late stage 5 embryos are in different phases of polarity formation.

### Characterization of genes identified in the stage 5 pRNAi and RNA-seq experiments

The 98 late stage 5 DEGs were categorized into four classes based on their responses to the *Pt-hh*/*Pt-ptc* pRNAi as follows: class I, down-/up-regulated; class II, up-/down-regulated; class III, down-/down-regulated; class IV, up-/up-regulated by the respective pRNAi. Expression patterns of all corresponding transcripts were examined in wild-type (WT) late stage 5 embryos by whole-mount in situ hybridization (WISH). These experiments demonstrated that approximately half of the genes (48 genes) had region-specific expression in the germ disc epithelium, and many of their expression domains exhibited concentric circular patterns (Fig. 2, C to E, table S2, and data file S5). Expression trends were correlated between the class of genes and the region of gene expression, although no specific expression patterns were detected for class IV genes. A total of 18 genes, of the 34 class I genes, were expressed in the peripheral region of the germ disc (e.g., *Pt-lab-A* expression; Fig. 2E), and 14 of the 23 class II genes were expressed in the central region (e.g., *Pt-msx1* and *Pt-gas1* expression; Fig. 2E). Further, 10 of the 26 class III genes were expressed in the intermediate region (e.g., *Pt-frizzled4* expression; Fig. 2E). Notably, most expression domains were diminished or expanded by *Pt-hh* and *Pt-ptc* pRNAi, as seen by the quantified responses of transcript expression levels (Fig. 2, C to E, table S2, and data file S5). Simultaneous detection of transcript expression of multiple genes by fluorescence in situ hybridization (FISH) revealed that the expression domains of these genes were abutted and overlapped, with peak areas located at varied distances from the germ disc rim, where *Pt-hh* is expressed (Fig. 2F). These expression patterns were reminiscent of the “French flag” (*12*), although with less clear spatial boundaries. These results indicated that a variety of cell states was specified by Hh signaling, according to their positions from the germ disc rim.

In addition, 10 genes from classes I to III were expressed at the cumulus (Fig. 2C, table S2, and data file S5), where the cumulus mesenchymal (CM) cells, a cell cluster that functions in dorsal-ventral axis formation, are migrating underneath the surface epithelial cells (*15*, *16*). Hh signaling was found to be involved in regulating the gene expression in the epithelial cells associated with the CM cells, as was evident from the effects of *Pt-hh* and *Pt-ptc* pRNAi on aug3.g17729 (*Pt-noggin-D*) expression (Fig. 2E). In contrast, altered Hh signaling did not seem to affect the gene expression in the CM cells (Fig. 2C and data file S5). Furthermore, *Pt-fgf8* is expressed rather stochastically, but the transcripts tended to be detected near the cumulus (Fig. 2E).

### Identification of *Pt-msx1* as a segmentation gene

Class I genes that were positively regulated by Hh signaling included *Pt-otd* (aug3.g9172) and *Pt-opa* (aug3.g12202). These genes have been previously characterized as Hh signaling targets that mediate stripe-splitting dynamics in head segmentation (*14*, *18*, *28*). In contrast, class II genes were negatively regulated by Hh signaling and have not been characterized previously. To investigate novel aspects of patterning mechanisms, we performed expression analysis of class II genes (fig. S2) and a pilot functional screen using pRNAi-mediated knockdown (*29*). From these analyses, we explored one class II gene aug3.g15189 (*Pt-msx1*), which encodes a homeodomain transcription factor and is a homolog of the *Drosophila muscle segment homeobox* (*msh*) gene and vertebrate *Msx* genes (*30*).

**Fig. 3:**
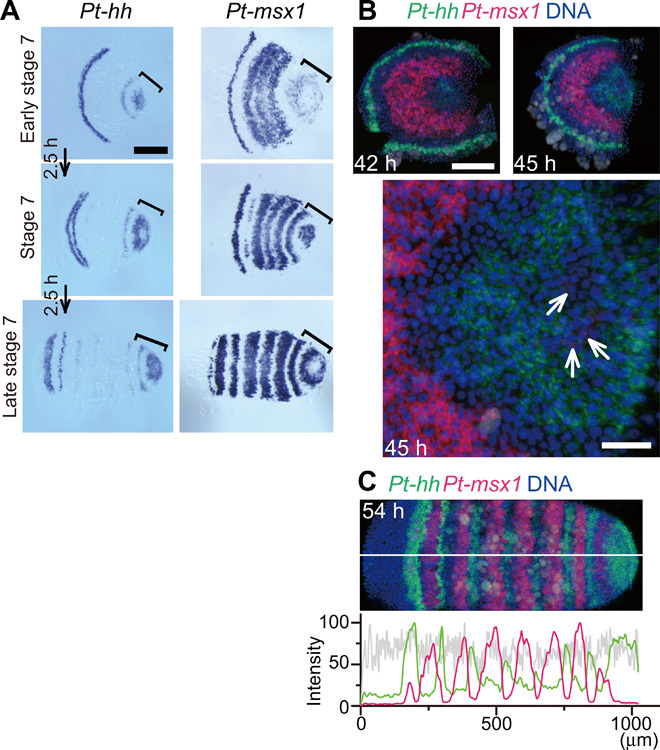
Complimentary striped expression of *Pt-msx1*and *Pt-hh*. (**A**) WT sibling embryos serially fixed at 2.5-hour intervals starting from early stage 7 and stained for *Pt-hh* and *Pt-msx1* transcripts. Brackets mark the opisthosomal region. (**B** and **C**) WT sibling embryos stained for *Pt-msx1* (red) and *Pt-hh* (green) transcripts and DNA (blue). Semi-flat preparations (B, top), the extracted surface image of the opisthosomal region of the 45-hour embryo (B, bottom), and the germ band of a 54-hour embryo (200-μm wide) (C). Signal intensity profiles in the surface cell layer along the midline (white line; averaged across 20 μm width) is shown together (C). White arrows indicate cells expressing *Pt-msx1* (B). Scale bars, 50 (B, bottom) and 200 μm (others).

Expression analysis of class II genes revealed *Pt-msx1* as one of the genes expressed in the central region of the germ disc at late stage 5 (fig. S2, A and B). Its expression was expanded to almost the entire region in the *Pt-hh*^pRNAi^ germ disc and was missing in *Pt-ptc*^pRNAi^ (Fig. 2E). Among class II genes, *Pt-msx1* and *Pt-ci* were the only genes that showed spatially repetitive stripes of expression in WT embryos by 50 hours after egg laying (AEL; fig. S2, A to C). In the presumptive opisthosomal region of the nascent germ bands, *Pt-msx1* expression exhibited a dynamic pattern similar to that of *Pt-hh* (Fig. 3A), indicating that it could behave as oscillatory traveling waves, whereas *Pt-ci* and most other class II genes did not (fig. S2). The complementary patterns of *Pt-hh* and *Pt-msx1* expression were observed at the earliest stage of oscillation (Fig. 3B) and were eventually established throughout the entire germ band (Fig. 3C).

**Fig. 4:**
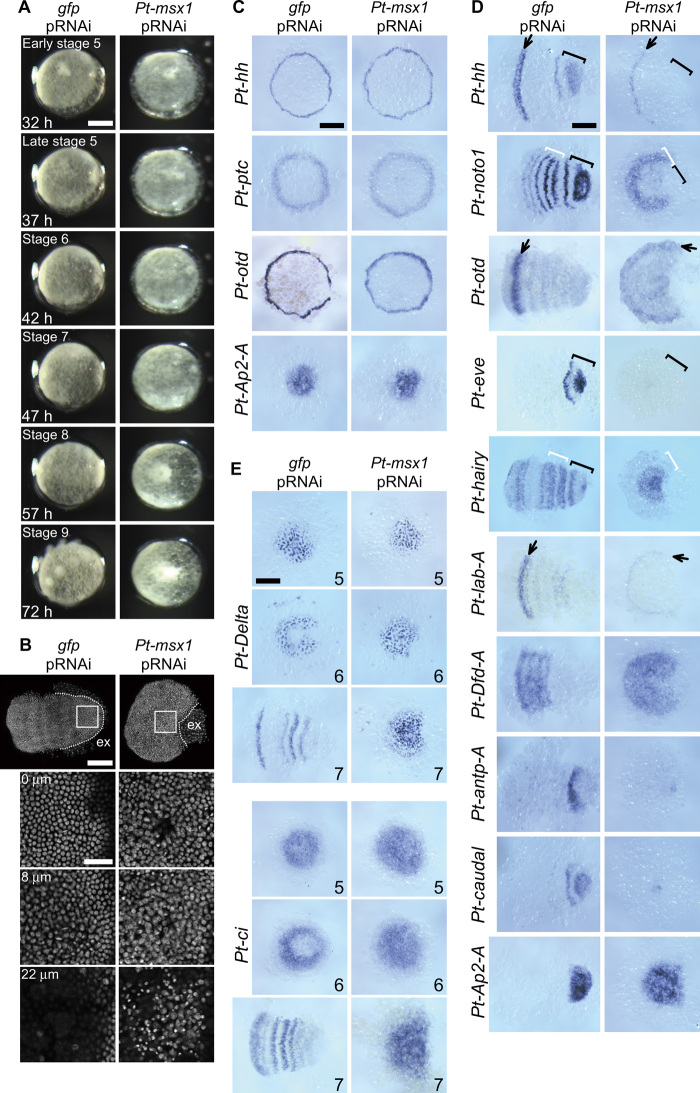
Effects of *Pt-msx1* pRNAi on periodic stripe formation. (**A**) Developmental time course of *gfp*^pRNAi^ and *Pt-msx1*^pRNAi^ embryos. These images are related to movie S1. Approximate times AEL and developmental stages are shown. The *Pt-msx1*^pRNAi^ embryo does not transform into a germ band, remaining in a fan-like shape. (**B**) *gfp*^pRNAi^ and *Pt-msx1*^pRNAi^ embryos at stage 7 stained for DNA. The boxed caudal regions are magnified in bottom panels, which show the images at three different depths: 0 μm, surface epithelial cell layer; 8 μm, mesodermal cell layer; 22 μm, yolk layer (*gfp*^pRNAi^) or interior cells, some of which appear to be dying (*msx1*^pRNAi^). The white dotted lines indicate the boundary between embryonic and extraembryonic (ex) area. (**C** to **E**) *gfp*^pRNAi^ and *Pt-msx1*^pRNAi^ embryos at late stage 5 (C), stage 7 (D), and stages indicated at the bottom right (E) stained for transcripts indicated. The *gfp*^pRNAi^ germ discs stained for *Pt-otd* and *Pt-AP2A* are the same as in data file S5. Arrows, the stripes in the head; white brackets, the thoracic region; black brackets, the opisthosomal region (D). The *Pt-msx1*^pRNAi^ embryos do not show repetitive striped expressions of genes (D and E). Anterior is to the left. Scale bars, 50 (B, bottom) and 200 μm (others). Photo credit: Yasuko Akiyama-Oda, JT Biohistory Research Hall.

Knockdown experiments revealed that *Pt-msx1* pRNAi led to failure to form germ bands and repetitive structures, despite the normal appearance of the germ disc (Fig. 4A and movie S1). *Pt-msx1*^pRNAi^ embryos initiated the differentiation of the extraembryonic region in response to the arrival of the CM cells at the germ disc rim but did not fully expand it and remained in a fan-like shape without elongating the emerging AP axis, even at the stage corresponding to the limb-forming stage (stage 9; Fig. 4A and movie S1). The caudal part of the embryonic region of *Pt-msx1*^pRNAi^ embryos started to show thickening after the beginning of stage 7; although the surface epithelial cells appeared morphologically normal, in the interior, some cells appeared to be dying (Fig. 4B). *Pt-msx1* pRNAi specificity was carefully confirmed (fig. S3), and the phenotypes of *Pt-msx1*^pRNAi^ embryos were then assessed at the molecular level. In *Pt-msx1*^pRNAi^ germ discs, the expression of *Pt-hh* and *Pt-ptc* and formation of AP polarity were relatively normal (Fig. 4C), in contrast to *Pt-hh*^pRNAi^ and *Pt-ptc*^pRNAi^ germ discs (Fig. 1A and data file S5). In the *Pt-msx1*^pRNAi^ embryos at later stages, the primary *Pt-hh* stripe traveled normally from the rim of the germ disc, but in the nascent germ bands, the stripe failed to enter the splitting phase, and the expression of *Pt-hh* and *Pt-otd* became obscure in the head (Fig. 4D). In the thorax of the nascent WT germ band, *Pt-noto1* and *Pt-hairy* are expressed in stripes (*17*, *31*). In the *Pt-msx1*^pRNAi^ embryos, *Pt-noto1* expression was detected in the putative thoracic region, disappearing from the most posterior part. In contrast, *Pt-hairy* expression was persistent in the posterior region. Both genes, however, did not show the spatially periodic expression (Fig. 4D). Notably, in the opisthosoma of the *Pt-msx1*^pRNAi^ embryos, oscillatory expression of genes—including *Pt-hh*, *Pt-noto1*, and a homolog of *Drosophila* pair-rule gene, *Pt-even-skipped* (*Pt-eve*)—did not occur (Fig. 4D). Moreover, serially arranged expression of *hox* genes, *Pt-lab-A*, *Pt-Dfd-A*, and *Pt-antp-A* (*25*), and *Pt-caudal* was not properly developed in *Pt-msx1*^pRNAi^ embryos (Fig. 4D); the anterior *Pt-lab-A* expression became reduced. The thoracic gene, *Pt-Dfd-A*, was expressed in the wide area of the embryonic region without showing any indication of stripes, while the expressions of the posterior genes, *Pt-antp-A* and *Pt-caudal*, were detected in only a few cells. In addition, *Pt-Ap2-A* (aug3.g23531) was expressed in a broad posterior region (Fig. 4D).

The normal germ disc displays a “salt-and-pepper” pattern of *Pt-Delta* expression at the central area reflecting opisthosomal mesoderm versus ectoderm fate decisions (*19*). As the germ disc transition toward the germ band, this *Pt-Delta* pattern disappears from the germ disc center, followed by segmental striped expression of *Pt-Delta* in the opisthosomal ectoderm (Fig. 1B and fig. S2D) (*14*, *19*). In *Pt-msx1*^pRNAi^ embryos, the salt-and-pepper pattern of *Pt-Delta* expression was normal but persistent and failed to shift to the segmental mode (Fig. 4E). Similarly, the expression pattern of *Pt-ci* was affected by *Pt-msx1* knockdown, with neither central attenuation nor transition to stripes (Fig. 4E). These observations suggested that *Pt-msx1* is not necessary for forming graded positional information in the cell field but is essential for generating spatial repetitions of gene expression.

### Regulation of periodic expressions of genes by *Pt-msx1*

**Fig. 5:**
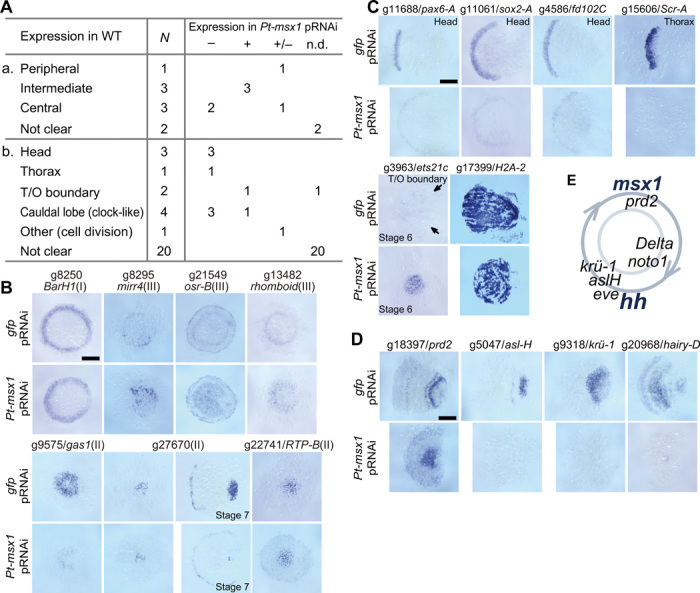
Striped and oscillatory expression of DEGs identified in *Pt-msx1* pRNAi and RNA-seq experiments. (**A**) Summary of expression of 40 selected DEGs: (a) nine DEGs that were also identified in *Pt-hh* and *Pt-ptc* pRNAi experiments; (b) other 31 DEGs. Expression areas in the epithelium of WT germ discs (a) and germ bands (b), numbers of DEGs (*N*), and expression in *Pt-msx1*^pRNAi^ embryos, where “–” indicates expression domains diminish, “+” indicates expression domains enlarge, and “+/−” indicates expression domains not clearly different from WT. T/O boundary, thorax-opisthosoma boundary. (**B** to **D**) *gfp*^pRNAi^ and *Pt-msx1*^pRNAi^ embryos stained for transcripts of genes that are listed in Aa (B) and Ab (C and D) (“not clear” genes were omitted). Embryos were at late stage 5 (B), stage 7 (C and D), or stages as indicated. The numbers in parentheses indicate the classes of the genes (B). Arrows (C) (aug3.g3963) point to the boundary between the thorax and opisthosoma, where very faint signals are observed. (**E**) Relative times of onsets of segmentation clock gene expression during the oscillation cycle at the posterior pole of the germ band. Anterior is to the left (stage 7). Scale bars, 200 μm. Photo credit: Yasuko Akiyama-Oda, JT Biohistory Research Hall.

To examine gene regulation by *Pt-msx1*, we performed RNA-seq analysis of *Pt-msx1*^pRNAi^ embryos, followed by WISH-based gene expression screening of 40 selected genes (Fig. 5A, table S3, and data file S6). These included nine genes that were also identified as DEGs in the *Pt-hh* and *Pt-ptc* pRNAi and RNA-seq experiments at late stage 5 (Fig. 5Aa). Of these nine genes, the intermediate expressions of three class III genes, aug3.g8295 (*Pt-mirr4*), aug3.g21549 (*Pt-odd-skipped related-B*), and aug3.g13482 (*Pt-rhomboid*), were expanded into the central region of the germ disc by *Pt-msx1* knockdown; in contrast, the expressions of two class II genes, aug3.g9575 (*Pt-gas1*) and g27670, were reduced within the central region (Fig. 5B). These results suggested that the germ disc patterning involved regulatory interactions among Hh signaling targets.

Other identified genes (Fig. 5Ab) included those expressed in stripes in the presumptive head [aug3.g11688 (*Pt-pax6-A*), aug3.g11061 (*Pt-sox2-A*), aug3.g4586 (*Pt-fd102C*)] or thorax [aug3.g15606 (*Pt-Scr-A*)] or at the boundary between the thorax and opisthosoma [aug3.g3963 (*Pt-ets21c*)]; the expressions of these genes diminished (the former four genes) or expanded to the posterior region (aug3.g3963) in *Pt-msx1*^pRNAi^ embryos (Fig. 5C). Four other genes, aug3.g18397 (*Pt-prd2*), aug3.g5047 (*Pt-asl-H*), aug3.g9318 [*Pt-krüppel-1* (*Pt-krü-1*)], and aug3.g20968 (*Pt-hairy-D*) exhibited cyclic expression patterns in the posterior terminal portion of the germ band (fig. S4A), as was the case with *Pt-hh* and *Pt-msx1* (Fig. 3A); these “segmentation clock” genes were not expressed or were static in the *Pt-msx1^pRNAi^* embryos (Fig. 5D). Simultaneous detection of expression of multiple clock genes in WT germ bands revealed differential phases of oscillations (fig. S4); *Pt-msx1* expression was antiphase with *Pt-hh* expression but was nearly in phase with *Pt-prd2* expression (Fig. 5E). Unlike in some insects and myriapods (*32*, *33*), none of these clock genes were expressed with double-segment periodicity in the spider. Collectively, these observations suggested that *Pt-msx1* functioned as a segmentation gene in all the three body regions of *Parasteatoda* embryos.

### Wave-like expression of *Pt-msx1* preceding stripe pattern formation

Next, we characterized events forming the striped expression of *Pt-msx1* at cellular resolution. Transcriptomic profiling by RNA-seq showed that *Pt-msx1*, similar to *Pt-hh*, started to be transcribed at stage 2 (12 to 14 hours AEL) (*26*). By FISH, *Pt-msx1* transcripts were not detectable at 24 hours AEL but were detected stochastically at 28 and 30 hours AEL among surface cells near the embryonic pole or the center of the nascent germ disc (fig. S5), which is on the opposite side of the egg relative to the position of initial *Pt-hh* transcription (*14*).

**Fig. 6:**
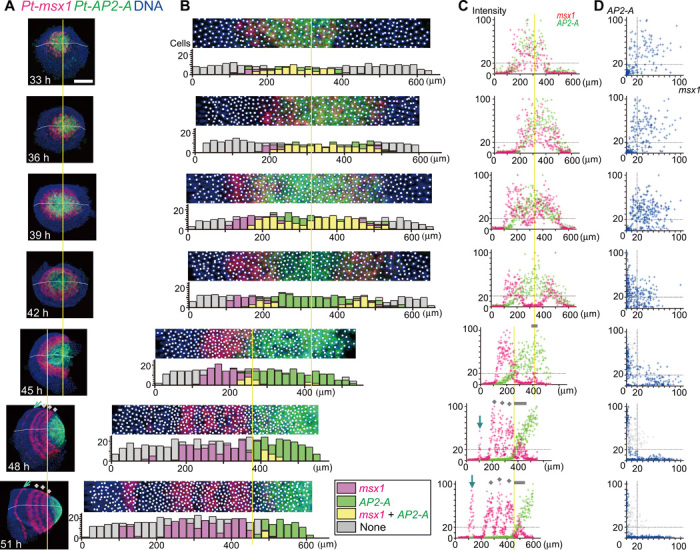
Dynamics of the primary wave of *Pt-msx1* expression. (**A** to **D**) Sibling embryos at the indicated time points stained for transcripts of *Pt-msx1* (red), *Pt-AP2-A* (green), and DNA (blue). (A) Semi-flat preparations. (B) Extracted images (80-μm wide) along the midline (white line, A). At the position of nuclei, white spots are placed, from where the quantitative signal values were extracted. Bar graphs show numbers of cells expressing substantial transcript levels (color-coded, inset) in each subdivided area (20 μm by 80 μm). (C) Dot plots showing relationships between cell positions and signal intensities of *Pt-msx1* (red) and *Pt-AP2-A* (green). (D) Dot plots showing relationships between signal intensities of *Pt-msx1* and *Pt-AP2-A* in individual cells. Gray spots (45 to 51 hours) (D) were derived from cells at the AP position range that are indicated by gray bars in (C), which mark *Pt-msx1* expression for the O1 segment. Yellow lines refer to the center of the germ disc and the likely corresponding position (33 to 45 hours) and the posterior limit of the *Pt-msx1* L4 stripe (45 to 51 hours). Gray dots, three *Pt-msx1* stripes (L2 to L4) resulting from tri-splitting of the primary *Pt-msx1* wave; blue arrows, *Pt-msx1* stripes at the bi-splitting sites (A and C). Anterior is to the left (36 to 51 hours). Germ disc orientation is arbitrary at 33 hours. Scale bar, 200 μm.

**Fig. 7:**
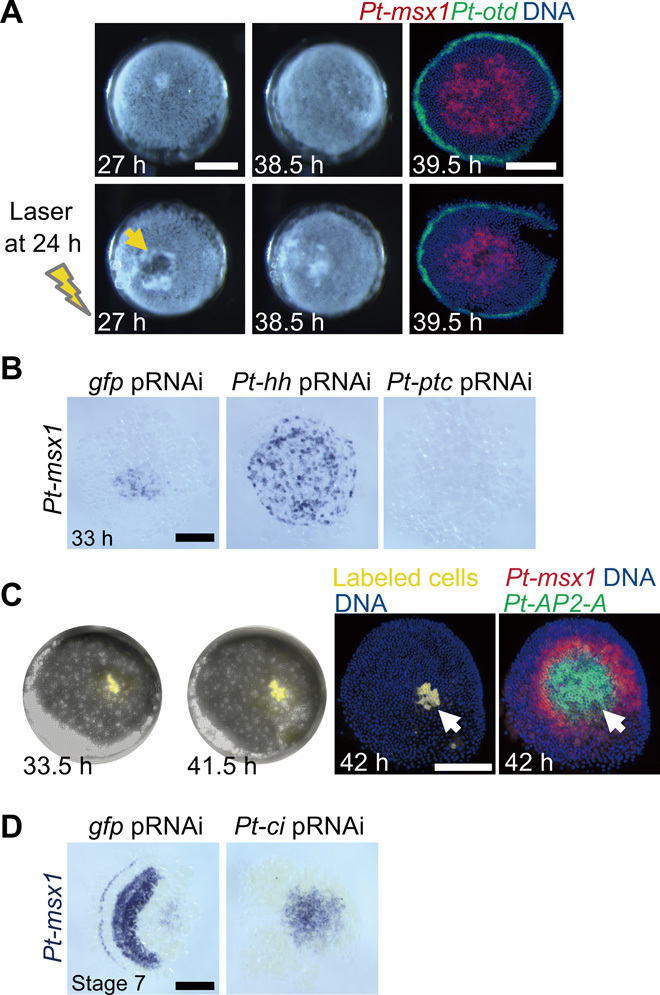
Propagation and attenuation dynamics of the *Pt-msx1* primary wave. (**A**) Initiation and propagation of the *Pt-msx1* wave independent of the central part of the early germ disc. An embryo was laser-irradiated to extirpate the region surrounding the embryonic pole (arrow) at 24 hours AEL and was photographed at 27 and 38.5 hours AEL together with an untreated sibling. Both embryos were fixed at 39.5 hours and were stained for *Pt-msx1* (red) and *Pt-otd* (green) transcripts and DNA (blue). (**B**) Early regulation of *Pt-msx1* expression by Hh signaling. *Pt-msx1* transcript staining of *gfp*^pRNAi^, *Pt-hh*^pRNAi^, and *Pt-ptc*^pRNAi^ germ discs at early stage 5. The expression already expanded to the periphery in the *hh*^pRNAi^ germ disc. (**C**) *Pt-msx1* expression dynamics in a static field. A live embryo with a labeled cell clone (yellow) was photographed at 33.5 and 41.5 hours AEL and was then stained as indicated. Arrows indicate the clone position, which remained largely unchanged, and the expression of *Pt-msx1* was detected in the more peripheral region. (**D**) Effects of *Pt-ci* knockdown on the *Pt-msx1* expression. *gfp*^pRNAi^ and *Pt-ci*^pRNAi^ embryos at stage 7 were stained for *Pt-msx1* transcripts. *Pt-msx1* continued to be expressed in the corresponding caudal region in the *Pt-ci*^pRNAi^ embryo. Anterior is to the left (D). Scale bars, 200 μm. Photo credit: Yasuko Akiyama-Oda, JT Biohistory Research Hall.

We then fixed sibling embryos, serially from the early germ disc stage, and first examined the spatial distributions of cells expressing *Pt-msx1*, relative to cells expressing another class II gene, *Pt-AP2-A* (aug3.g23531; Fig. 6 and fig. S5B). Observations from these experiments revealed three successive phases of *Pt-msx1* expression. During the first phase, cells expressing these two genes were initially found only near the embryonic pole but covered up to more than half of the diameter of the germ disc, by 36 hours AEL. Extirpation of the embryonic polar region by laser irradiation at 24 hours AEL failed to prevent expansion of *Pt-msx1* expression in cells in the broad central area of the later germ disc (Fig. 7A), suggesting that *Pt-msx1* expression is independent of the potential signaling center at the embryonic pole (*34*). In contrast, *Pt-ptc* knockdown completely abolished *Pt-msx1* expression (Figs. 2E and 7B), and *Pt-hh* knockdown caused overexpansion of *Pt-msx1*–expressing cells even at earlier germ disc stages (33 hours AEL; Fig. 7B). Hence, expansion dynamics of *Pt-msx1* expression were regulated by Hh signaling.

During the second phase, *Pt-msx1*, and not *Pt-AP2-A*, expression started to be attenuated in cells in the central area of the germ disc (39 hours; Fig. 6). Cells expressing both *Pt-msx1* and *Pt-AP2-A* decreased in number (42 hours) and more peripheral (anterior) cells exhibited *Pt-msx1*–biased expression. As revealed by cell labeling experiments, *Pt-msx1* expression was attenuated in a static field of cells (Fig. 7C). This indicated that a large-scale wave of *Pt-msx1* expression developed from around the embryonic pole, which corresponded to the future posterior pole, and swept toward the thorax. Notably, the cells in which the *Pt-msx1* expression was attenuated initiated *Pt-hh* expression (Fig. 3B). Furthermore, *Pt-ci* knockdown embryos were defective in the central attenuation of *Pt-msx1* expression (Fig. 7D), as in the case with *Pt-ci* expression in *Pt-msx1* knockdown embryos (Fig. 4E). These observations suggested that *Parasteatoda* embryonic segmentation involved mutually opposing regulations of *Pt-msx1* and Hh signaling through gene networks that have not yet been characterized. Similar wave-like behaviors of gene expression patterns, starting from around the embryonic pole in the germ disc stage, were observed for eight more class II genes and the *Pt-Delta* gene (*14*, *19*), with varied timing and expansion range (fig. S2, A and D). Here, these early wave-like gene expressions were referred to as the primary waves. With remodeling of the field into a germ band, the *Pt-msx1* and *Pt-AP2-A* expression domains were spatially separated, with the exception of a de novo *Pt-msx1* peak for the first opisthosomal (O1) segment (45 to 51 hours; Fig. 6).

During the third phase, the primary *Pt-msx1* wave, after passing through the future opisthosoma and arriving at the future thorax, split into three stripes in a short time frame (48 and 51 hours; Fig. 6). These stripes corresponded to anterior portions of the second to fourth leg-bearing thoracic (L2 to L4) segments. This “tri-splitting” can be compared with “bi-splitting” of the *Pt-hh* stripe during head segmentation (*18*), but the former was temporally nonrepetitive, and the latter was temporally repetitive.

**Fig. 8:**
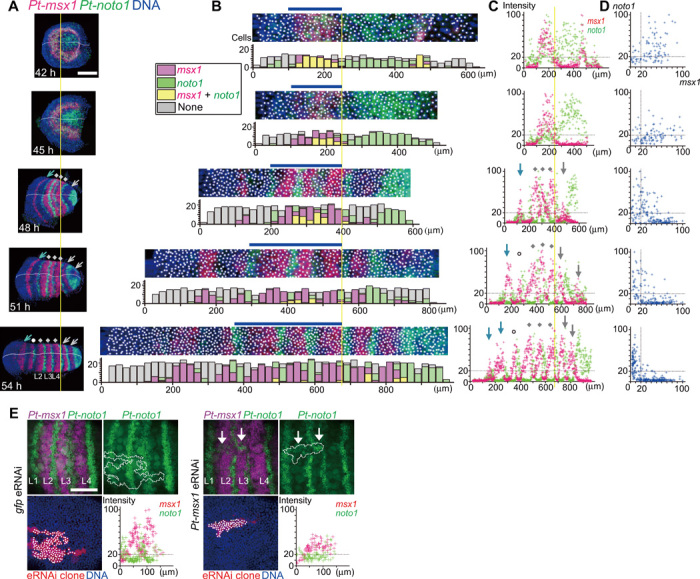
Tri-splitting dynamics of the *Pt-msx1* expression. (**A** to **D**) Sibling embryos at the indicated time points stained for *Pt-msx1* (red) and *Pt-noto1* (green) transcripts and DNA (blue). Data and images are shown as in Fig. 6. In (D), intensity values were extracted from cells at the position range indicated by blue bars in (B). Yellow lines refer to the posterior limit of the *Pt-msx1* L4 stripe. Gray dots, three *Pt-msx1* stripes (L2 to L4) resulting from tri-splitting of the primary *Pt-msx1* wave; gray arrows, *Pt-msx1* expression peaks or stripes for O1 and O2 segments generated through oscillations; blue arrows, *Pt-msx1* stripes at the bi-splitting sites; white circles, the *Pt-msx1* L1 stripe appearing later (A and C). (**E**) Phenotypes of *Pt-msx1*^eRNAi^ and *gfp*^eRNAi^ cell clones during tri-splitting. The samples were stained for *Pt-msx1* (magenta) and *Pt-noto1* (green) transcripts, eRNAi cell clones (red), and DNA (blue). Dot plots show relationships between positions of cells (spots) along the AP axis and signal intensities of *Pt-msx1* (red) and *Pt-noto1* (green) in the eRNAi cell clones (outlined). White arrows indicate disordered patterns of *Pt-noto1* expression in the *Pt-msx1*^eRNAi^ cell clone. Anterior is to the left. Scale bars, 200 μm (A) and 100 μm (E).

We reconstructed the tri-splitting process by staining sibling embryos for *Pt-msx1* and *Pt-noto1* (Fig. 8, A to D, and fig. S6). *Pt-noto1* is one of the earliest indicators of thoracic segmentation (Fig. 1B) (*17*) and is expressed ubiquitously in the presumptive thoracic region of the germ disc in early embryos (fig. S2D). In this reconstruction, the *Pt-msx1* primary wave merged with *Pt-noto1* expression in the thoracic field, and, subsequently, complementary striped distribution of *Pt-msx1*– and *Pt-noto1*–expressing cells was generated, as double-positive cells disappeared. Considering previously described cell tracking data (*17*), this tri-splitting was likely elicited through transcriptional regulation in the epithelium, with limited contribution from cell sorting. To confirm the roles of *Pt-msx1* in tri-splitting, we introduced *Pt-msx1* embryonic RNAi (*Pt-msx1*^eRNAi^) cell clones into the presumptive thoracic region. These clones locally impaired the patterning event (15 of 17), whereas *gfp*^eRNAi^ cell clones did not (0 of 10; Fig. 8E). *Pt-msx1*^eRNAi^ cell clones did not develop spatially organized, complementary expressions of *Pt-msx1* and *Pt-noto1*. These data confirmed the functional importance of the *Pt-msx1* wave in thoracic segmentation.

### The onset of oscillatory expression in the caudal region

**Fig. 9:**
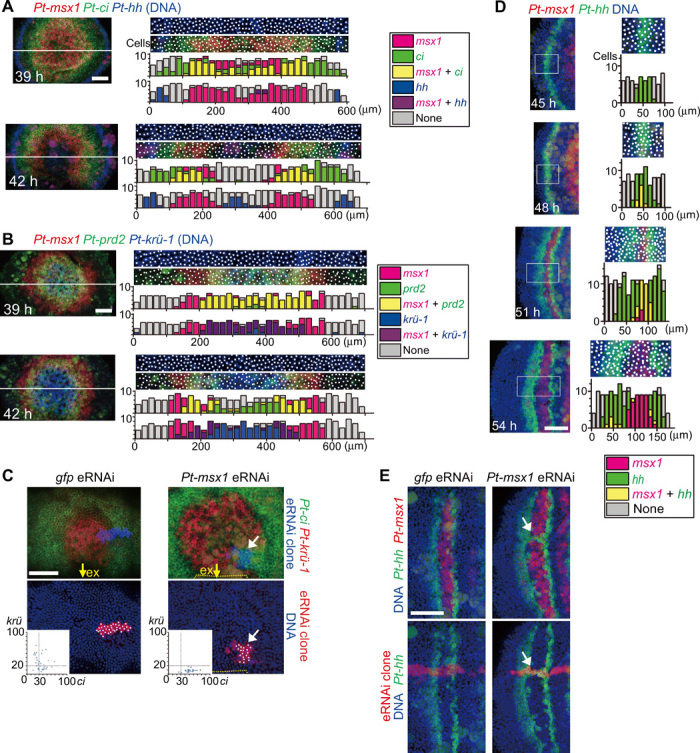
*Pt-msx1*–mediated distinct segmentation dynamics in different body regions. (**A** and **B**) Initiation of clock gene expression. Sibling embryos at 39 and 42 hours AEL were stained for the indicated transcripts and DNA (blue). Extracted images (50-μm wide) along the line (left) are shown with bar graphs that show numbers of cells (spots) expressing substantial transcript levels (color-coded, inset) in each subdivided area (20 μm by 50 μm). (**C**) Phenotypes of *Pt-msx1*^eRNAi^ and *gfp*^eRNAi^ cell clones in the opisthosomal region at early stage 6. The samples stained for *Pt-ci* and *Pt-krü-1* transcripts, eRNAi cell clones, and DNA are shown as indicated. Dot plots (insets) show signal intensities of *Pt-ci* and *Pt-krü-1* in individual cells (spots) in the cell clones. Yellow arrows, the extraembryonic (ex) region; yellow dotted lines, the boundary between embryonic and ex areas (*Pt-msx1*^eRNAi^). The *Pt-msx1*^eRNAi^ embryo is slightly older than the *gfp*^eRNAi^ embryo, but most cells in the *Pt-msx1*^eRNAi^ cell clone (white arrows) remained expressing *Pt-ci* and not *Pt-krü-1*. (**D**) Insertion of *Pt-msx1* expression between the splitting *Pt-hh* stripes in the head region. The head region of sibling embryos stained for *Pt-msx1* (red) and *Pt-hh* (green) transcripts and DNA (blue). The boxed 80-μm-wide regions were extracted, and bar graphs were produced as in (A) and (B), except that each subdivided area is 10 μm by 80 μm. (**E**) Phenotypes of *Pt-msx1*^eRNAi^ and *gfp*^eRNAi^ cell clones during bi-splitting. The samples stained for *Pt-hh* and *Pt-msx1* transcripts, eRNAi cell clones, and DNA are shown as indicated. White arrows mark unseparated *Pt-hh* stripes. Anterior is to the top (A to C) and to the left (D and E). Scale bars, 100 μm.

The field through which the primary *Pt-msx1* wave passed initiated cyclic expression of segmentation clock genes (fig. S4). In particular, the propagation and attenuation dynamics of the primary *Pt-msx1* expression wave were closely associated with the attenuation of *Pt-ci* expression and the initiation and propagation of expression of serial clock genes, including *Pt-hh*, *Pt-prd2*, and *Pt-krü-1* (Fig. 9, A and B). All these expression dynamics were affected in *Pt-msx1*^pRNAi^ embryos (Figs. 4 and 5). To confirm the roles of *Pt-msx1* in regulating these dynamics, we examined *Pt-msx1*^eRNAi^ cell clones that were introduced to the central region of the germ disc. These cell clones, but not *gfp*^eRNAi^ cell clones, prevented both *Pt-ci* attenuation and *Pt-krü-1* initiation (6 of 6; 0 of 5 for *gfp*^eRNAi^; Fig. 9C). In the representative *Pt-msx1*^eRNAi^ cell clone, most cells remained *Pt-ci* positive and did not initiate *Pt-krü1* expression despite the progression of *Pt-ci* attenuation outside of the cell clone (Fig. 9C). This was in contrast with the *gfp*^eRNAi^ cell clone, which included cells expressing *Pt-ci*, *Pt-krü1*, or both according to the progression of *Pt-ci* attenuation (Fig. 9C). These results suggested that the primary *Pt-msx1* wave is among the key mechanisms that underlie the initiation of the genetic oscillations associated with opisthosomal segmentation. In addition, as mentioned above, *Pt-msx1* attenuation was abolished in *Pt-ci*^pRNAi^ embryos (Fig. 7D), suggesting that interdependent negative regulation of *Pt-msx1* and Hh signaling is a prerequisite for initiating the patterning cycles.

### Regulation of bi-splitting dynamics by *Pt-msx1* in the head segmentation

In the presumptive head region, temporally repeated bi-splitting of *Pt-hh* stripes gives rise to head segments (*18*). Expression analysis revealed a nascent stripe of *Pt-msx1* expression in the head region (Figs. 6 and 8), and the *Pt-msx1*^pRNAi^ embryos exhibited reduced expressions of head genes (Figs. 4D and 5C). Simultaneous detection of *Pt-hh* and *Pt-msx1* expression in sibling embryos revealed that *Pt-msx1* was dynamically expressed in association with the bi-splitting of *Pt-hh* stripes (Fig. 9D). In particular, cells expressing *Pt-msx1* were observed within the *Pt-hh* stripe preceding each bi-splitting event, and *Pt-msx1* expression expanded to occupy the space between the splitting stripes. We found that *Pt-msx1*^eRNAi^ cell clones failed to abolish *Pt-hh* expression between the splitting stripes (15 of 15; 0 of 9 for *gfp*^eRNAi^; Fig. 9E). *Pt-msx1*^eRNAi^ cell clones did not induce *Pt-hh* expression outside of the *Pt-hh* stripes. These findings suggested that *Pt-msx1* acts as a negative regulator of *Pt-hh* expression to mediate the generation of bi-splitting dynamics for head segmentation.

## DISCUSSION

**Fig. 10:**
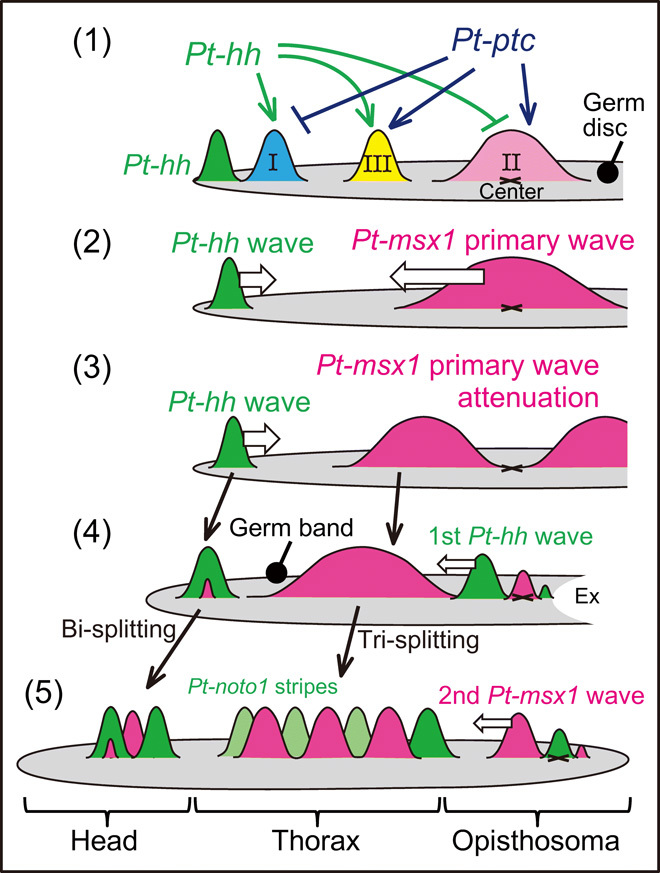
Schematic diagram of early patterning in the spider embryo. Germ-disc patterning and varied stripe-forming events were controlled by Hh signaling along the radius of the germ disc or along the AP axis of the germ band. Expressions of class I, II, and III genes in the germ disc are regulated by *Pt-hh* and *Pt-ptc*. The class II gene *Pt-msx1* is involved in generating distinct gene expression dynamics in the three body regions. See the main text for details.

The present genome-wide analyses of *P. tepidariorum*, using a combination of *Pt-hh* and *Pt-ptc* pRNAi and RNA-seq, have provided comprehensive evidence that Hh signaling mediates the formation of a polarized French flag–like pattern in a cell-based field of the germ disc in the spider embryo (Fig. 2). This pattern comprised concentric circular expressions of class I to III genes, which were achieved through differential transcriptional responses to the initial Hh signaling activities [Fig. 10 (1)]. Transcription of class I genes was positively regulated by Hh signaling, that of class II genes was negatively regulated, and that of class III genes required both *Pt-hh* and *Pt-ptc*. *Pt-hh* is expressed at the periphery of the germ disc corresponding to the future anterior (Figs. 1 and 2). The radius of the germ disc is about 300 μm, which is comparable to the effective range of Hh molecules shown in vertebrate studies (*35*, *36*). Although any protein products of Hh signaling components have not been examined in the spider embryos, our previous observation regarding the changing pattern of *Pt-ptc* transcript expression (*14*), which is generally considered to be the indicator of Hh signal reception (*7*), supports the notion that the Hh signaling network operates in the full spatial range of the spider germ disc. Our extensive gene expression analyses combined with the gene knockdown experiments revealed the direct involvement of Hh signaling in generating spatially organized diverse cell states in the entire germ disc before the emergence of spatially periodic patterns.

The French flag–like pattern in the spider germ disc seems to be comparable with the expression pattern of *Drosophila* gap genes in the blastoderm, which is regulated by the graded concentration of Bicoid (*37*). Despite the differences in the nature of the patterning fields (syncytial versus cellular) and in the type of the master molecules (the transcription factor, Bicoid, versus the secreted signaling protein, Hh), similar expression patterns of a large spectrum of genes are displayed in both static fields. At first, *Drosophila* gap gene expression domains overlap, but sharp boundaries are established depending on mutually repressive interactions between gap genes (*37*). In the spider, clear boundaries of gene expression domains were not observed in the germ-disc stage (Fig. 2), but interactions among target genes appeared to occur. For example, the expression patterns of some of class II and class III genes were reduced and expanded, respectively, by knockdown of a class II gene, *Pt-msx1* (Fig. 5). The periodic stripe pattern of *Drosophila* pair-rule genes develops in the static field (*20*). In contrast, spatial repetition in the spider embryo became apparent only after transition of the germ disc toward the germ band began (Figs. 1, 6, and 8).

A prominent aspect of Hh signaling–based patterning, which is directly associated with periodic stripe formation, was revealed by our findings on the expression and function of the transcription factor gene *Pt-msx1*, which is a class II gene. As summarized in Fig. 10, *Pt-msx1* exhibited expression associated with three types of waves and mediated the formation of spatially repeated stripes in all the body regions. Hence, *Pt-msx1* is a key mediator of the initial Hh signaling activity and downstream patterning system. The primary *Pt-msx1* wave arose from around the center of the germ disc (corresponding to the posterior pole of the later embryo) [Fig. 10 (2)], propagated to the future thoracic region in the nascent germ band [Fig. 10 (3 and 4)], and then split into three stripes complementary to those of *Pt-noto1* (tri-splitting) [Fig. 10 (4 and 5)], thereby specifying the thoracic segments [Fig. 10 (5)]. In the future opisthosomal region, where the primary *Pt-msx1* wave passed away due to attenuation, oscillatory expressions of segmentation clock genes, including *Pt-hh* and *Pt-msx1*, followed, which temporally behaved as traveling waves [Fig. 10 (4 and 5)]. In the future head region, the *Pt-hh* stripe initiated at the rim of the germ disc traveled posteriorly and underwent two rounds of bi-splitting, each of which is preceded by an onset of *Pt-msx1* expression in the *Pt-hh* stripe [Fig. 10 (2) to (5)]. Collectively, the dynamics of *Pt-msx1* expression under the control of Hh signaling are distinct depending on the body regions of the spider embryo, and all are related to segmentation.

The primary expression waves of *Pt-msx1* and certain class II genes started around the site corresponding to the posterior pole and then propagated toward the future thorax (fig. S2A). These wave propagations were concurrent with that of *Pt-Delta*, which was described in the previous studies (*14*, *19*). This type of phenomenon, where expression of negatively regulated targets of Hh signaling starts at the side opposite to the Hh signaling source in a large field and dynamically propagates in the field, has not been reported in other organisms. The laser ablation experiment suggested that cells at and around the expression start site made little contribution to the expansion of *Pt-msx1* expression (Fig. 7A). Instead, involvement of Hh signaling in the regulation was suggested by the pRNAi experiments (Figs. 2E and 7B). Our previous model, explaining the expansion dynamics of *Pt-Delta* expression, incorporated the role of *Pt-ptc* in altering the effective distance of Hh molecules from longer to shorter (*14*) and may be applicable to the expanding expressions of class II genes. However, because neither Hh protein distribution nor Hh receptor behavior has been examined, the ideas remain hypothetical. For the attenuation dynamics, data from *Pt-ci* and *Pt-msx1* knockdown suggested the presence of mutually repressive interactions between *Pt-msx1* and Hh signaling (Figs. 4E, 7D, and 9C). Overall, the dynamic response and wave-like behavior of the Hh signaling activities that occur in the surface epithelial cell sheet of the germ disc and germ band are easily accessible, and the simple features of the spider embryonic field may be advantageous for the study of Hh signaling–mediated patterning dynamics.

The tri-splitting of the primary *Pt-msx1* wave presented the segmentation dynamics in the thoracic region described in this study (Figs. 6 and 8). The requirement of *Pt-msx1* for this phenomenon was shown by the eRNAi experiment (Fig. 8E). Although expression of *Pt-hh* transcripts in this region started later than tri-splitting, it appeared at the cells expressing *Pt-noto1* and eventually formed a complementary pattern to the *Pt-msx1* stripes (Fig. 3). Ahead of, or simultaneous with, the propagation of the *Pt-msx1* wave, the thoracic field displayed a French flag–like pattern, which was likely to be under the control of Hh signaling (Fig. 2). It is possible that genes expressed to form the French flag–like pattern contribute to the tri-splitting phenomenon cooperatively with *Pt-msx1*. Although the *Pt-hairy* gene, described in a previous study (*31*), presents expression dynamics rather similar to those of *Pt-msx-1* in the thoracic and opisthosomal regions, its expression pattern development was found to depend on *Pt-msx1* (Fig. 4D). Interactions between Hh signaling downstream activities should be addressed in future studies to better understand the Hh signaling–based patterning systems.

Oscillation is the segmentation dynamics observed in the opisthosomal region. FISH staining showed that expressions of multiple clock genes, including *Pt-msx1* and *Pt-hh*, repeatedly oscillated in the cells of the opisthosoma, exhibiting repeated wave-like propagation in one segment periodicity in this opisthosomal field (fig. S4). The first oscillatory waves followed the primary waves for *Pt-msx1* and other class II genes that propagated toward the thorax, and they did not occur in the *Pt-msx1* knockdown embryos (Figs. 4, 5, and 9). Oscillation is a common strategy to establish a spatially periodic stripe pattern, as seen in somitogenesis of vertebrates and segmentation of short germ insects (*20*, *33*). However, the initiation mechanisms for the first oscillatory waves had been rarely studied. The spider embryo thus provides a unique opportunity to analyze the onset of oscillation. Moreover, the clock genes identified in the *Pt-msx1* pRNAi and RNA-seq experiments were family genes of a *Drosophila* gap gene (*Pt-krü-1*), a pair-rule gene (*Pt-prd2*), and basic helix-loop-helix–type vertebrate oscillatory genes (*Pt-hairy-D* and *Pt-asl-H*) (Fig. 5). Studies of spider segmentation may contribute to filling the gap in our understanding of the periodic stripe-forming mechanisms among animals.

Further, we unexpectedly found that *Pt-msx1* was involved even in the repeated bi-splitting of *Pt-hh* stripes, associated with the head segmentation (Fig. 9). It is most likely that *Pt-msx1* acts cooperatively with region-specific factors for head segmentation because *Pt-msx1* knockdown cell clones prevented the down-regulation of *Pt-hh* expression between the splitting *Pt-hh* stripes but did not induce ectopic *Pt-hh* expression outside the areas of the splitting stripes. The candidate genes include *Pt-otd* and *Pt-opa*, which have been shown to play roles in the bi-splitting event (*18*, *28*). Interactions of *Pt-msx1* with these genes are subjects of future studies.

Collectively, our findings have demonstrated that Hh signaling controls all the segmentation dynamics via *Pt-msx1* in a spider embryo. *Pt-msx1* was found to be one of the most sensitive genes to the altered states of Hh signaling in the germ disc stage, and mutually exclusive expressions and repressive interactions between *Pt-msx1* and Hh signaling components were observed during the bi-splitting and at the onset of the oscillatory waves. We suggest a deep origin of involvement of *msx1* and *hh* in segmentation because striped expression of a *msx1* homolog is conserved in harvestman embryos (*30*) and that of *hh* homologs is in a wide range of arthropods (*14*, *22*, *23*). This study provides a new paradigm, beyond the *Drosophila*-based knowledge framework, of segmentation and lays the foundation for comparative investigations of segmentation mechanisms across the arthropod phylum.

Evolvability of Hh signaling–based patterning systems has attracted increasing attention in terms of animal form diversification (*38*–*41*). Mathematical models have previously proposed that a minimal mechanism of Hh signaling coupled with a regulatory network of transcription factors confers various pattern-forming capabilities (*42*). This proposal might be potentially applicable to spider embryos. It is required to investigate whether *Pt-msx1* cooperates with region-specific transcription factor genes to achieve segmentation diversity.

## MATERIALS AND METHODS

### Spider egg collection

The animal experimentation was conducted according to the protocol reviewed and approved by the Institutional Animal Care and Use Committee of JT Biohistory Research Hall (2005-5). We used laboratory stocks of the spider *P. tepidariorum* (synonym, *A. tepidariorum*), which were kept at 25°C with a 16-hour light/8-hour dark cycle. Developmental stages have been described previously (*15*). Developmental time AEL was estimated from the beginning of stage 2 (10 hours AEL; the nuclei reach the surface of the egg) or the beginning of stage 5 (30 hours AEL; internalization of the CM cells is completed). All mated females were evaluated by monitoring development of their embryos to exclude data derived from nonreliable embryos; morphology of germ discs at stage 5 and that of germ bands at stages 7 and 9 was examined. In experiments using earlier embryos, development of their siblings was examined. In pRNAi experiments (see below), embryos were checked whether they form morphologically normal germ discs.

We prepared 12 independent (derived from different egg sacs) groups of sibling embryos fixed at multiple time points. These embryo groups were used for (i) Figs. 1B, 3 (B and C), 8A, and 9 (A, B, and D), and fig. S4 (B and C); (ii) Fig. 3A and fig. S4A; (iii) fig. S5A; (iv) Fig. 6 and fig. S6; (v) fig. S4 (D to F); (vi) fig. S5B; and (vii to xii) fig. S2. The embryos shown in fig. S2 were derived from a total of seven egg sacs and were examined by *Pt-msx1* staining to confirm that the developmental stages were consistent among them.

### RNA interference

dsRNAs were synthesized using a MEGAscript T7 Transcription kit (Ambion, AMB13345) as described previously (*16*). *At-hh1*, *At-ptc1*, and *At-ci1* dsRNAs (*14*, *18*) were used for pRNAi against *Pt-hh*, *Pt-ptc*, and *Pt-ci*, respectively. The *gfp* dsRNA was used as a negative control. For pRNAi against *Pt-msx1*, *Pt-msx1-*1 and *Pt-msx1-*2 dsRNAs were synthesized from nonoverlapping regions (nucleotides 1 to 351 and 352 to 737) of the expressed sequence tag (EST) clone *At_eW_020_B15* (LC379630.1). In pRNAi experiments, dsRNA was injected at a concentration of 2.0 μg/μl. All females were given four injections of dsRNA solution (1 to 2 μl each) at 2- to 3-day intervals. Spiders lay eggs repeatedly every about 5 days, and the effect of dsRNA injections usually appears in the eggs laid after 10 days from the first injection and in the peak around 20 days after the first injection. Eggs that were laid at 13 days or later from the day of the first dsRNA injection were used as pRNAi samples.

To verify the specificity of RNAi for *Pt-msx1*, we confirmed that injections of *Pt-msx1*-1 and *Pt-msx1*-2 dsRNAs produced the same morphological phenotypes with complete penetrance. The defects of transition to the germ band were observed in 7 of 7 females injected with *Pt-msx1*-1 dsRNA and in 3 of 3 females injected with *Pt-msx1*-2 dsRNA (fig. S3A). We also confirmed that the same gene expression phenotypes were obtained with both dsRNAs in WISH analyses of the transcripts shown in Figs. 4 and 5. In this paper, the presented *Pt-msx1* knockdown data, including those of RNA-seq, were generated using *Pt-msx1*-1 dsRNA unless stated otherwise.

eRNAi experiments were performed as previously described (*18*, *28*) using *msx1*-1 dsRNA and *gfp* dsRNA. Briefly, a mixture of dsRNA (0.4 μg/μl), Rhodamine B isothiocyanate (RITC)–dextran [0.8 μg/μl; weight-average molecular weight (*M*_w_) of 70,000; Sigma-Aldrich, R9379], and biotin-dextran (0.8 μg/μl; *M*_w_ of 10,000; Sigma-Aldrich, B9139) was microinjected into single blastomeres of 32- or 64-cell stage embryos, which were then allowed to develop until stage 6 or 7 and were then fixed for staining. RITC-dextran was used to visualize injected cell clones in living embryos, whereas biotin-dextran was used to visualize cell clones in the fixed samples.

### RNA sequencing

Poly(A) RNA was extracted from 50 to 120 embryos at early stage 3, late stage 5, and early stage 6. These embryos were produced by single females before dsRNA injection (the first egg laying) and at 13 to 24 days after the first dsRNA injection. DEGs were identified from comparisons of the former control samples and the latter test (pRNAi) samples. Comparative sets of RNA-seq libraries were generated for *Pt-hh*, *Pt-ptc*, and *gfp* pRNAi-treated embryos at early stage 3 and late stage 5 and for *Pt-msx1* and *gfp* pRNAi-treated embryos at late stage 5 and early stage 6. These libraries were prepared with two or three biological replicates (different females). Poly(A) RNA extraction was performed using the QuickPrep Micro mRNA Purification Kit (GE Healthcare) or the Dynabeads mRNA DIRECT Micro Kit (Veritas, DB61012). mRNA samples were quantified using the Qubit fluorometric RNA HS assay (Life Technologies, Q32852), and whole amounts (20 to 50 ng) were used in subsequent steps. The mRNA was fragmented with the New England Biolabs (NEB)Next RNase III RNA Fragmentation module (NEB, E6146), and samples were then used for RNA-seq library construction using the NEBNext Ultra Directional RNA Library Prep Kit for Illumina (NEB, E7420) and the NEBNext Multiplex Oligos for Illumina (Index Primers Set 1; NEB, E7335). Quantitative PCR were performed using a KAPA or Takara Library Quantification kit (KAPA, 4824; Takara, Z8324N). Libraries were then sequenced in single-end runs in the antisense direction using the MiSeq reagent kit V3 (150 cycles; Illumina, MS-102-3001) on an Illumina MiSeq platform. Between 12 and 24 million reads were generated for each library. Information for constructed libraries and RNA-seq reads is summarized in data file S1.

### Sequence read trimming, mapping on the genome, and quantification

Raw reads of the MiSeq sequencing were trimmed in three steps using the CLC Genomics Workbench (v. 7.0.4). Adapter trimming, sequence primer trimming, and quality trimming with the following parameter settings: trim using quality scores, limit of 0.05; trim ambiguous nucleotides, maximum number of ambiguities 2; and filter on length, discard reads below length 50. Trimmed reads were mapped onto the *P. tepidariorum* genome sequence assembly Ptep_1.0 (GCA_000365465) using the alignment tool BLAT (v.34) (*43*) in the DDBJ Read Annotation Pipeline with default settings. Output alignments were filtered on the basis of quality, coverage, and uniqueness using a perl script filterPSL.pl, which is available from the AUGUSTUS 3.0.1 scripts folder (https://github.com/Gaius-Augustus/Augustus). The parameter settings were as follows: minimum coverage, 60%; minimum percent identity, 90%; and unique threshold, 0.96. Filtered alignments that were in the pslx format were then converted into the psl and sam formats with a perl script written in our laboratory. Using sam files, mRNA abundances against AUGUSTUS gene models (aug3) (*25*) were calculated using htseq-count v.0.5.4p5 (*44*) with -s reverse, -m union settings. The results of trimming, mapping, and htseq-counts are summarized in data file S1, and counts per gene are listed in data file S2. Mapping results were visualized in University of Tokyo Genome Browsers (*45*) using wig files that were converted from the psl files with a script aln2wig, which is available from the AUGUSTUS 3.0.1 scripts folder, and were normalized as counts per 10 million reads.

### Identification of DEGs

We used the R software (v. 3.1.2) edgeR_3.8.5 (*46*) to identify DEGs. In these analyses, pairwise comparisons were performed with datasets from the same parent pair so that pseudo-positives due to individual variations were reduced. Genes with very low counts (less than 1 count per million reads in all samples) were filtered out before running edgeR. Genes with the adjusted *P* value (FDR) < 0.01 were listed as DEGs after excluding genes with FDR < 0.01 in experiments of *gfp* pRNAi and RNA-seq at the same developmental stage. In DEG detection analyses using *Pt-hh* and *Pt-ptc* pRNAi samples, EdgeR was run with comparisons between control samples and (i) test samples derived from eggs laid at days 13 to 17 after the first injection of dsRNA, (ii) test samples derived from eggs laid at days 18 to 22, and (iii) all test samples. Genes that were identified as differentially expressed (FDR < 0.01) in any of the comparisons were all considered as DEGs (data files S3 and S4), and genes identified as DEGS in both *Pt-hh* and *Pt-ptc* pRNAi were selected for further analyses (table S2).

In *Pt-msx1* pRNAi experiments, we listed genes with FDR < 0.01 as DEGs (data file S6). In the stage 6 *Pt-msx1* pRNAi experiments, we selected a total of 40 DEGs based on the following two criteria: (i) genes with FDR < 0.01 in both *Pt-hh* and *Pt-ptc* pRNAi experiments at stage 5 (nine genes) and (ii) genes with the 31 lowest FDR values in *Pt-msx1* pRNAi experiments (FDR < 0.0003; table S3). We examined expression in germ disc epithelial cells for genes identified in i and in early germ bands for genes identified in ii (Fig. 5).

### cDNA cloning

Full-length or partial cDNAs of identified DEGs were obtained from our laboratory stocks of EST clones (*19*) or were isolated in PCR. The resulting cDNAs were used to synthesize probes. The cDNA clones and the PCR primers were listed in table S4.

### Live observations of embryogenesis

Embryos were prepared as previously described (*19*). Images were acquired every 10 min using a stereomicroscope SZX12 (Olympus) equipped with a color 3CCD (charge-coupled device) camera C7780-10 (Hamamatsu Photonics) and were processed using ImageJ version 1.47n.

### Cell labeling

A mixed solution of RITC-dextran (0.8 μg/μl; Sigma-Aldrich, R9379) and biotin-dextran (0.8 μg/μl; Sigma-Aldrich, B9139) was injected into single blastomeres of 32- or 64-cell stage embryos as described previously (*18*). Positions of labeled cell clones in germ discs of living embryos were recorded using fluorescence of RITC-dextran. Images were acquired using an Olympus BX50 fluorescence microscope equipped with a cooled CCD camera (CoolSNAP HQ; Roper Scientific) controlled by MetaMorph version 7.1 (Universal Imaging) and were processed using ImageJ version 1.47n. Embryos were then fixed at stage 6 for staining.

### Laser ablation

Laser ablation was performed as described previously (*34*). Briefly, dechorionated eggs were aligned on double sticky tape and were covered with halocarbon oil 700 (Sigma-Arldrich). Using an upright microscope (BX50, Olympus) equipped with the XYClone 20× laser objective (Hamilton Thorne), cells around the blastopore of the aligned embryos were heat-killed by irradiating with 13 to 22 laser pulses (1460 nm, 300 mW × 2 ms) per embryo. The embryo shown in the bottom panels of Fig. 7A was subjected to 16 pulses at about 24 hours AEL (late stage 3), thus affecting a region of about 10 cells in diameter.

### In situ hybridization and image acquisition

WISH and FISH were performed as described previously (*15*, *28*). Digoxigenin (DIG)–labeled probes were used for the alkaline-phosphatase chromogenic staining of WISH. In the staining shown in Fig. 3A and figs. S2 and S4A, signals were amplified with a combination of anti–DIG-POD (horse-radish peroxidase) (used at 1:1000 dilution; Roche, 11 207 733 910), dinitrophenyl (DNP)–tyramide (1:100 dilution; TSA plus DNP system, PerkinElmer), and anti–DNP-AP (1:100 dilution; Vector MB-3100). In FISH, DIG, fluorescein (Flu), and DNP probes were used in combination with DyLight488, AlexaFluor568, DyLight594, and DyLight680 tyramides. In cell labeling and eRNAi experiments, biotin-dextran introduced in cell clones was visualized using Cy5-streptavidin in fixed samples (GE Healthcare, PA45001). Most samples were counterstained with 4′,6-diamidino-2-phenylindole (DAPI; Sigma-Aldrich) to visualize DNA.

Stained embryos were mounted on glass slides with spacers. WISH samples were observed using a stereomicroscope system as described above (SZX12 and C7780-10) and an Axiophoto 2 fluorescence microscope (Zeiss) equipped with an AxioCam HRc (Zeiss). FISH samples were observed using a TCS SPE confocal system (Leica).

### Analysis of expression domains of DEGs in germ discs

To categorize expression domains in germ discs, the late stage 5 germ disc was roughly divided into three areas along the radius (about 25 cells) according to the head-thorax and thorax-opisthosoma fate boundaries at early stage 5 (*17*) as follows: the peripheral area, 1st to 8th cells from the periphery; the intermediate area, 5th to 15th cells; and the central area, from 13th cells to the center. Among 98 identified DEGs from late stage 5 *Pt-hh* and *Pt-ptc* pRNAi and RNA-seq experiments, 48 were relatively clearly expressed in localized regions of the germ-disc epithelium (Fig. 2 and data file S5), and their expression domains were categorized in the above criteria (Fig. 2, C and D, and table S2). Expressions were also examined at the cumulus. Because WISH signals for other genes were very weak, obscure, or ubiquitous or were detected in likely endoderm cells, we did not analyze their expressions further.

### Processing and analysis of FISH images

Z-series stacks of confocal images were processed using Imaris version 7.6.5 (Bitplane), ImageJ version 1.47n, and Adobe Photoshop CS6 and CC 2019 software. Low-magnification fluorescent images displayed in Figs. 1B, 2F, 3B, 6A, and 8A, and figs. S5B and S6 were produced using the snapshot function of Imaris software.

To count cell numbers in the surface cell layer (Fig. 1B), spots were placed on signals of the DAPI channel of the original confocal images using the Imaris automatic spot-detection function and manual correction. Spots were then counted. To generate intensity profiles of gene expression, measurement points were manually placed on the surface cell layer along the midline using the DAPI channel as a spatial reference. Along the three-dimensional (3D) midline connecting these measurement points, intensity values were calculated at every 1 μm as the mean across a width of 20 μm using bilinear interpolation with an ImageJ plugin as previously described (*17*). The extracted 1D intensity data were analyzed using OriginPro 2018J b9.5.0.193 (OriginLab); the intensity data, except those for the DAPI channel, were smoothened using the weighted adjacent-averaging method with a moving 11-point window and were then normalized in 0 to 100 between the minimum and maximum values. The intensity data for the DAPI channel were only subjected to normalization. Using a function of the ImageJ plugin mentioned above (*17*), flat images were extracted from 3D data along the specified line.

Extracted flat images were analyzed to obtain cell-level intensity values for gene expression. Using the Imaris automatic spot-detection function followed by manual correction, spherical spots of 5 μm in diameter were placed in the surface cell layer using the DAPI channel as a spatial reference, and intensity means within the sphere of the spots were extracted as intensity values of the cells. Values were normalized (0 to 100) between minimum and maximum values using OriginPro. Signal values in small areas (Figs. 8E and 9, A to D) were normalized using intensity data from the whole surface cell layer of 200-μm-wide extracted images. Normalized intensity values above 20 were considered substantial.

## SUPPLEMENTARY MATERIALS

Supplementary material for this article is available at http://advances.sciencemag.org/cgi/content/full/6/37/eaba7261/DC1

## Appendix

View/request a protocol for this paper from *Bio-protocol*.
